# PD-1/PD-L1 inhibitors monotherapy vs. combination therapy in elderly advanced NSCLC: a real-world study and nomogram for survival prognosis

**DOI:** 10.1186/s12890-025-03791-x

**Published:** 2025-07-23

**Authors:** Yunye Mao, An Wang, Xiangwei Ge, Jinzhao Zhai, Yi Hu, Jinliang Wang

**Affiliations:** 1 Senior Department of Oncology, the, Fifth Medical Center , PLA General Hospital, Beijing, 100853 China; 2 Chinese PLA Medical School, Beijing, 100853 China; 3 Department of Oncology, the, First Medical Center , PLA General Hospital, Beijing, 100853 China

**Keywords:** Elderly patients, Advanced non-small cell lung cancer, Immunotherapy, Immune combination therapy

## Abstract

**Background:**

Immunotherapy with PD-1/PD-L1 inhibitors has transformed advanced non-small cell lung cancer (NSCLC) treatment, yet optimal strategies for elderly patients remain uncertain. Elderly patients (≥ 65 years) exhibit immune senescence (e.g., T-cell dysfunction, chronic inflammation), which may compromise immunotherapy efficacy and amplify toxicity risks, yet direct comparisons of monotherapy versus combination regimens in this population are lacking. Real-world comparisons of monotherapy versus combination therapy in this vulnerable group are lacking, hindering personalized clinical decisions.

**Objective:**

This real-world study aimed to compare the efficacy and safety of PD-1/PD-L1 inhibitor monotherapy versus combination therapy with chemotherapy in elderly patients (≥ 65 years) with advanced NSCLC and develop a prognostic nomogram to guide personalized treatment decisions.

**Methods:**

In this multicenter retrospective study, 641 patients (149 monotherapy, 492 combination therapy) with stage IIIB/IV NSCLC were analyzed. Primary endpoints included overall survival (OS), progression-free survival (PFS), and adverse events (AEs). A nomogram incorporating clinical variables was constructed using LASSO-Cox regression.

**Results:**

In a retrospective analysis of 641 elderly patients (≥ 65 years) with advanced NSCLC, combination therapy (*n* = 492) demonstrated superior median OS compared to monotherapy (*n* = 149) (35.37 vs. 20.53 months; HR = 0.62, 95% CI 0.48–0.80, *P* < 0.001), though PFS did not differ significantly (11.87 vs. 10.67 months; HR = 0.94, *P* = 0.535). Age-stratified analysis revealed marked OS benefits for patients < 75 years receiving combination therapy (36.10 vs. 18.67 months, *P* < 0.001), whereas no advantage was observed in those ≥ 75 years (29.23 vs. 34.93 months, *P* = 0.645). Cox regression identified combination therapy as a protective factor (HR = 0.54, *P* < 0.001), while ECOG PS ≥ 2 (HR = 1.87, *P* = 0.002), liver metastasis (HR = 1.62, *P* = 0.013), bone metastasis (HR = 1.84, *P* < 0.001), and malignant pleural effusion (HR = 1.64, *P* < 0.001) independently worsened prognosis. The incidence of AEs of any-grade (*P* < 0.001) and grade 3–4 (*P* = 0.003) in the immunotherapy combination group was significantly higher than that in the immunotherapy monotherapy group. A prognostic nomogram integrating treatment type, ECOG PS score, and other six variables had an AUC value of 0.70–0.71 for predicting 1–2 year OS.

**Conclusions:**

For elderly patients with advanced NSCLC, immune combination therapy improved median OS over monotherapy. It was safe and effective, suggesting a viable treatment option, though further evaluation is needed for those aged 75 and older. A prognostic nomogram for OS following immunotherapy was developed, showing superior accuracy.

## Introduction

Lung cancer is a prevalent and deadly disease worldwide, with non-small cell lung cancer (NSCLC) being the most commonly diagnosed type. The elderly, 65 years and older, account for approximately 70% of newly diagnosed NSCLC patients[1, 2]. The treatment landscape for advanced NSCLC has seen a significant shift with the introduction of immune checkpoint inhibitors (ICIs), specifically anti-programmed cell death protein-1 (PD-1) and its ligand, programmed cell death ligand-1 (PD-L1), in combination with chemotherapy, which become the main option for advanced NSCLC without gene mutations, and has shown synergy with chemotherapy[3, 4]. Despite this, the evidence supporting the use of ICIs in elderly patients is not yet sufficient [5]. Keynote 024 and 042 studies have demonstrated the superiority of immune monotherapy over chemotherapy when PD-L1 expression is ≥ 50% [6, 7]. However, it has since been shown that NSCLC patients receiving immune combination chemotherapy as first-line therapy have better outcomes than chemotherapy alone, regardless of the level of PD-L1 expression[8–11]. While subgroup analyses of trials like KEYNOTE-189 suggest potential benefits of ICIs in elderly patients [12–15], real-world data specific to this population—especially those with comorbidities or frailty—remain sparse.

With age, elderly patients exhibit immune senescence in the form of epigenetic changes, loss of protein homeostasis, and cellular senescence, accompanied by reduced organ system function and the development of a variety of comorbidities, which result in lower response rates and reduced tolerance to conventional treatments such as chemotherapy, radiotherapy, and targeted therapy [16, 17] . Furthermore, adding chemotherapy to ICIs may increase treatment-related toxicity in older individuals, which is something to be concerned about. Consequently, only elderly patients were included in this study to assess the efficacy and safety disparities between immune monotherapy and immune combined with chemotherapy in elderly individuals diagnosed with advanced NSCLC.

## Methods and materials

### Study design

This multicenter retrospective study focused on advanced NSCLC patients aged 65 years and above, defined according to the World Health Organization's (WHO) criteria, admitted to the First, Third, and Fifth Medical Centers of the PLA General Hospital between January 2014 and April 2021. The patients were divided into two groups: the immunotherapy monotherapy group received ICIs monotherapy (monotherapy group), while the immune combined with chemotherapy group was treated with ICIs combined with chemotherapy (immune combination group). This study evaluated the effectiveness and safety of the monotherapy group and combination therapy group in elderly patients with advanced NSCLC. This research aimed to provide valuable insights into the optimal treatment approaches for this group of patients. This study was approved by the Ethics Committee of the PLA General Hospital (No. 2023–8–26–1), with informed consent waived by the Institutional Review Board of the PLA General Hospital.

### Patient selection criteria

The inclusion criteria are as follows: (1) NSCLC confirmed by histology or cytology; (2) Patients with stage IIIB or IV NSCLC according to the 8th edition of the TNM staging system of the American Joint Committee on Cancer (AJCC); (3) At least one measurable lesion according to RECIST 1.1; (4) Age 65 years or older; (5) First-line treatment with immunotherapy or immunotherapy combination; (6) At least two cycles of treatment completed and having baseline and ≥ 1 follow-up imaging evaluations; (7) Complete medical records and follow-up information.

The exclusion criteria are as follows: (1) Positive for other mutations such as epidermal growth factor receptor (EGFR) and anaplastic lymphoma kinase (ALK); (2) Patients with other primary malignancies or severe heart, brain, kidney diseases, and mental disorders; (3) Incomplete medical records.

### Study assessments

This study utilized RECIST 1.1 criteria to evaluate patient outcomes. The size of the target lesion was measured to assess maximum tumor shrinkage and determine imaging remissions, which included patients with complete response (CR), partial response (PR), or stable disease (SD). Overall survival (OS) was defined as the time from treatment initiation to the date of death from any cause or the date of the last follow-up. Progression-free survival (PFS) was defined as the time from treatment initiation to disease progression or death, whichever occurred first. Follow-up was conducted until April 30, 2023. Disease control rate (DCR) was defined as the proportion of patients with CR plus PR plus SD. Objective response rate (ORR) is defined as the proportion of patients with CR plus PR. Adverse events (AEs) were documented through safety assessments at baseline, as well as during follow-up visits or clinical evaluations. AEs were considered treatment-related based on assessments made by the attending physician. All treatment-related AEs were graded according to the common terminology criteria for adverse events (CTCAE) version 5.0.

### Statistical analysis

Statistical analysis was conducted utilizing SPSS 26.0 statistical software, and GraphPad Prism was employed for data visualization. Cohort characteristics were compared between the monotherapy group and the combination therapy group using standard statistical descriptors. Median OS and PFS were estimated using the Kaplan–Meier method and a 95% confidence interval (CI). Differences between the two treatment groups were assessed using the Log-rank test. The Cox proportional hazards regression model was utilized to determine the hazard ratio (HR) and 95% CI. A significance level of *P* < 0.05 indicated a statistically significant difference. Key predictive variables were selected using LASSO regression, and a nomogram prediction model was constructed using R 4.2.2 software. The performance of the model was evaluated through receiver operating characteristic (ROC) curves and calibration curves. Internal validation was conducted by using the modeling data itself to verify the predictive effect of the model. Additionally, decision curve analysis (DCA) was performed to determine the threshold of net benefit for prediction.

## Results

### Patient characteristic

By the end of the follow-up, a total of 641 patients with advanced NSCLC were included in this study. Among them, 149 patients received immunotherapy alone (monotherapy group), with a median age of 69 years (range: 65–88 years). 492 patients received immunotherapy combined with chemotherapy (immune combination group), with a median age of 68 years (range: 65–83 years). Regarding the ECOG PS score, 130 patients (87.25%) in the monotherapy group had a score between 0 and 1, while 19 patients (12.75%) had a score of ≥ 2. In contrast, 466 patients (94.72%) in the immune combination group had a score between 0 and 1, and only 26 patients (5.28%) had a score of ≥ 2 (*P* = 0.003). (Table 1).

**Table 1:** Baseline and treatment characteristics

Characteristics	Monotherapy Group n = 149	Immune Combination Group n = 492	*P* value
Age (Median)	69 (65–91)	68(65–87)	0.117
Sex, n (%)			0.126
Male	117 (78.52%)	413 (83.94%)	
Female	32 (21.48%)	79 (16.06%)	
Age, n (%)			0.077
< 75	115 (77.18%)	411 (83.54%)	
≥ 75	34 (22.82%)	81 (16.46%)	
BMI, n (%)			0.076
< 24.8	94 (63.09%)	270 (54.88%)	
≥ 24.8	55 (36.91%)	222 (45.12%)	
ECOG PS, n (%)			0.002
0–1	130 (87.25%)	466 (94.72%)	
≥ 2	19 (12.75%)	26 (5.28%)	
Smoking Status, n (%)			0.829
Never	52 (34.90%)	167 (33.94%)	
Former/Current	97 (65.10%)	325 (66.06%)	
Histological, n (%)			0.544
Squamous	76 (51.01%)	237 (48.17%)	
Non-Squamous	73 (48.99%)	255 (51.83%)	
Stage, n (%)			0.161
IIIB	42 (28.19%)	169 (34.35%)	
IV	107 (71.81%)	323 (65.65%)	
Metastasis, n (%)			0.161
No	42 (28.19%)	169 (34.35%)	
Yes	107 (71.81%)	323 (65.65%)	
Liver Metastases, n (%)			0.621
No	134 (89.93%)	449 (91.26%)	
Yes	15 (10.07%)	43 (8.74%)	
Bone Metastases, n (%)			0.861
No	111 (74.50%)	370 (75.20%)	
Yes	38 (25.50%)	122 (24.80%)	
Brain Metastases, n (%)			0.558
No	136 (91.28%)	441 (89.63%)	
Yes	13 (8.72%)	51 (10.37%)	
Pleural Effusion, n (%)			0.126
No	126 (84.56%)	388 (78.86%)	
Yes	23 (15.44%)	104 (21.14%)	
PD-L1			0.005
< 1%	10 (6.71%)	65 (13.21%)	
1%−49%	21 (14.09%)	110 (22.36%)	
≥ 50%	28 (18.79%)	60 (12.20%)	
未知	90 (60.40%)	257 (52.24%)	
Complication, n (%)			0.414
No	82 (55.03%)	252 (51.22%)	
Yes	67 (44.97%)	240 (48.78%)	

*BMI * Body Mass Index, *ECOG PS* Eastern Cooperative Oncology Group Performance Status, *PD-L1* programmed cell death ligand 1

### Efficacy

In the immune combination therapy group (*n* = 149), CR was achieved in 1 case (0.67%), PR in 36 cases (24.16%), SD in 81 cases (54.36%), and PD in 31 cases (20.81%). In contrast, the monotherapy group (*n* = 492) showed CR in 4 cases (0.81%), PR in 258 cases (52.44%), SD in 200 cases (40.65%), and PD in 30 cases (6.10%). (Table 2).

**Table 2:** Evaluation of efficacy

	**Monotherapy Group** **n = 149**	**Immune combination Group** **n = 492**
Tumor response		
ORR	37 (24.83%)	262 (53.25%)
DCR	118 (79.19%)	462 (93.90%)
Best overall response		
CR	1 (0.67%)	4 (0.81%)
PR	36 (24.16%)	258 (52.44%)
SD	81 (54.36%)	200 (40.65%)
PD	31 (20.81%)	30 (6.10%)

*ORR* objective response rate, *DCR* disease control rate, *CR* complete response, *PR* partial

**Fig. 1:**
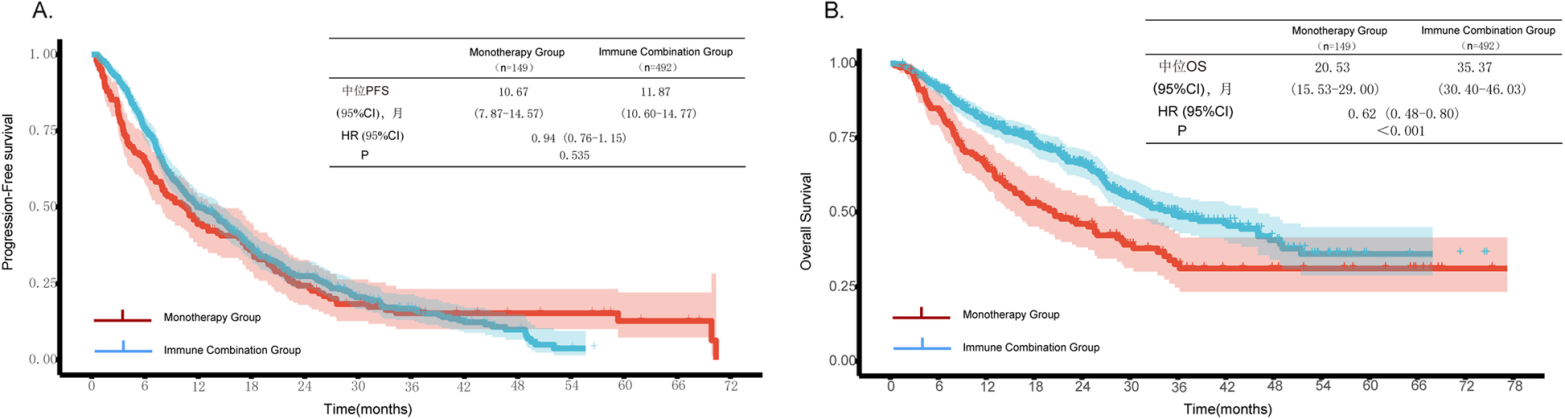
Kaplan–Meier curves showing (**A**) PFS between ICIs monotherapy and ICIs combined with chemotherapy, and (**B**) OS between ICIs monotherapy and ICIs combined with chemotherapy

In terms of PFS, the median PFS of the immune combination group was 11.87 months (95%CI 10.60–14.77), and that of the monotherapy group was 10.67 months (95%CI 7.87–14.57). No significant difference was observed between the two groups (HR = 0.94, 95CI 0.76–1.15, *P* = 0.535) (Fig. 1A). Regarding OS, the median OS of the immune combination group was 35.37 months (95%CI 30.40–46.03), significantly longer than that of the monotherapy group, which was 20.53 months (95%CI 15.53–29.00). The risk of death in the immune combination group was significantly reduced by 38% (HR = 0.62, 95%CI 0.48–0.80, *P* < 0.001) (Fig. 1B).

### Subgroup analysis

**Fig. 2:**
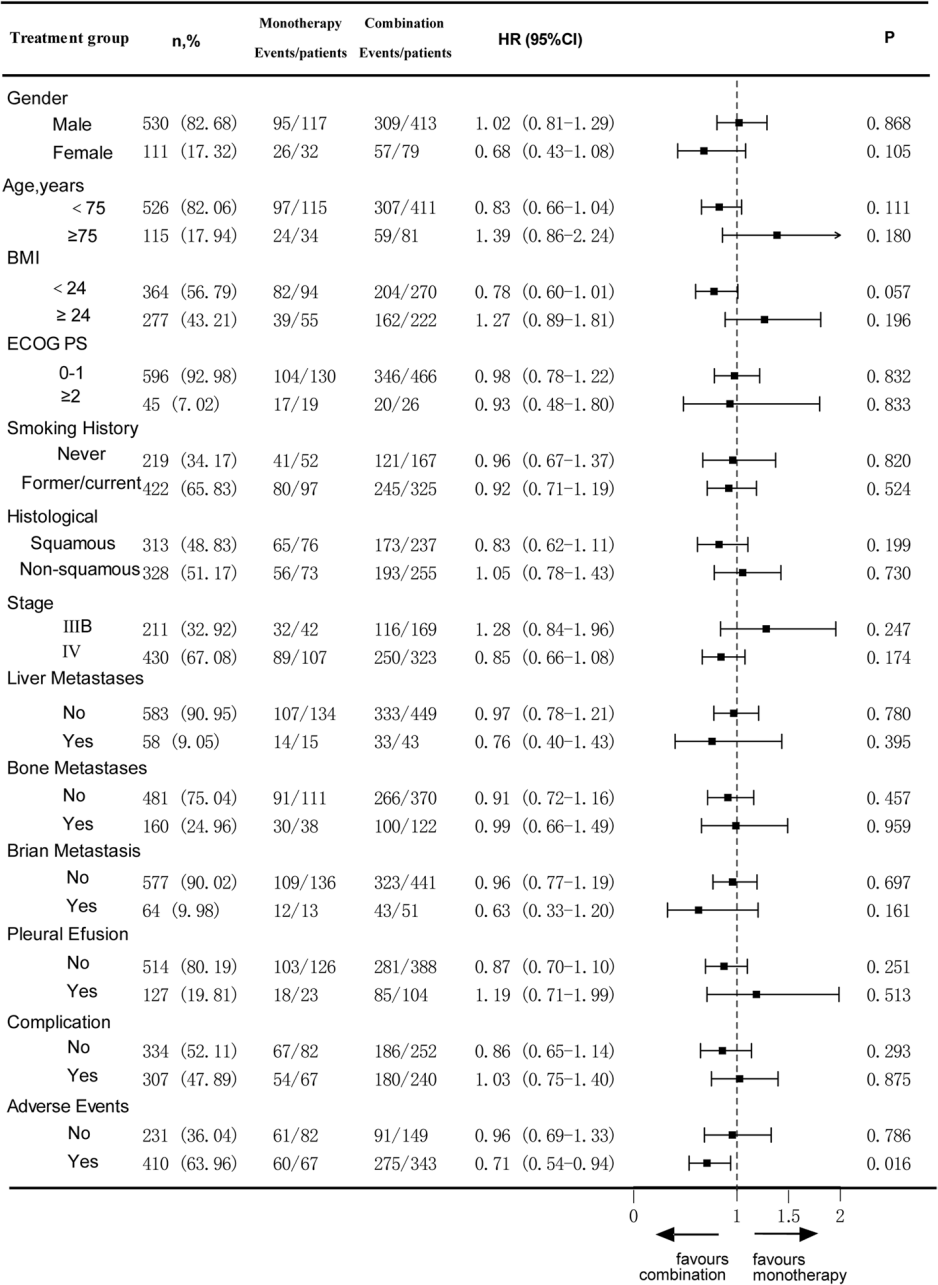
Subgroup analysis of PFS based on different characteristics

This study conducted a subgroup analysis to evaluate the impact of immune monotherapy versus combination therapy on PFS and OS in patients with different characteristics. In the subgroup analysis for PFS, patients who only experienced AEs (HR = 0.71, 95%CI 0.54–0.94, P = 0.016) showed better efficacy in the immune combination group. Additionally, patients with age < 75 years (HR = 0.83, 95%CI 0.66–1.04, P = 0.111) and BMI < 24 kg/m^2^ (HR = 0.78, 95CI 0.60–1.01, P = 0.057) also demonstrated a trend of benefit (Fig. 2).

**Fig. 3:**
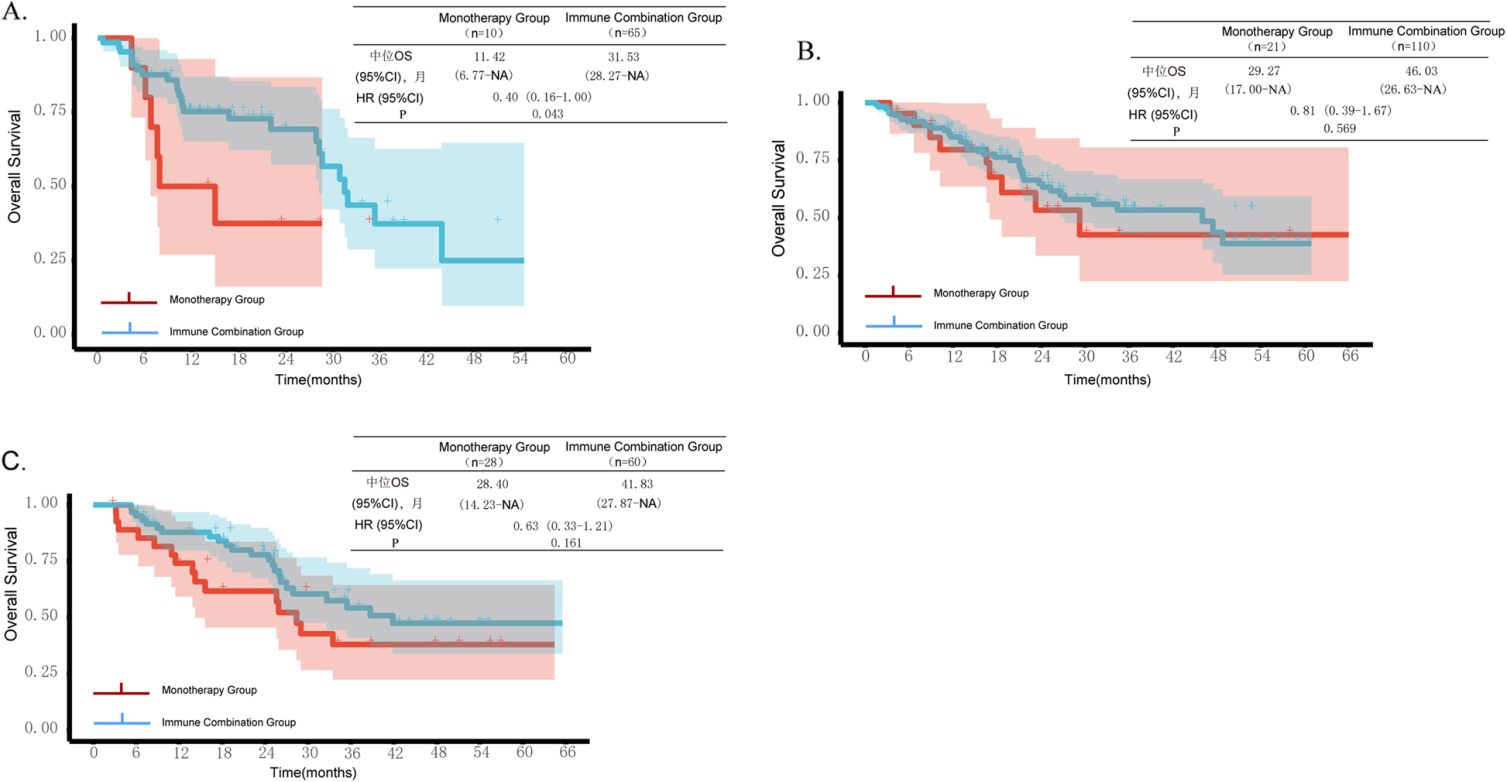
Kaplan–Meier curves showing (**A**) OS in patients with PD-L1 expression < 1%, (**B**) OS in patients with PD-L1 expression between 1%−49%, and (**C**) OS in patients with PD-L1 expression ≥ 50%

**Fig. 4:**
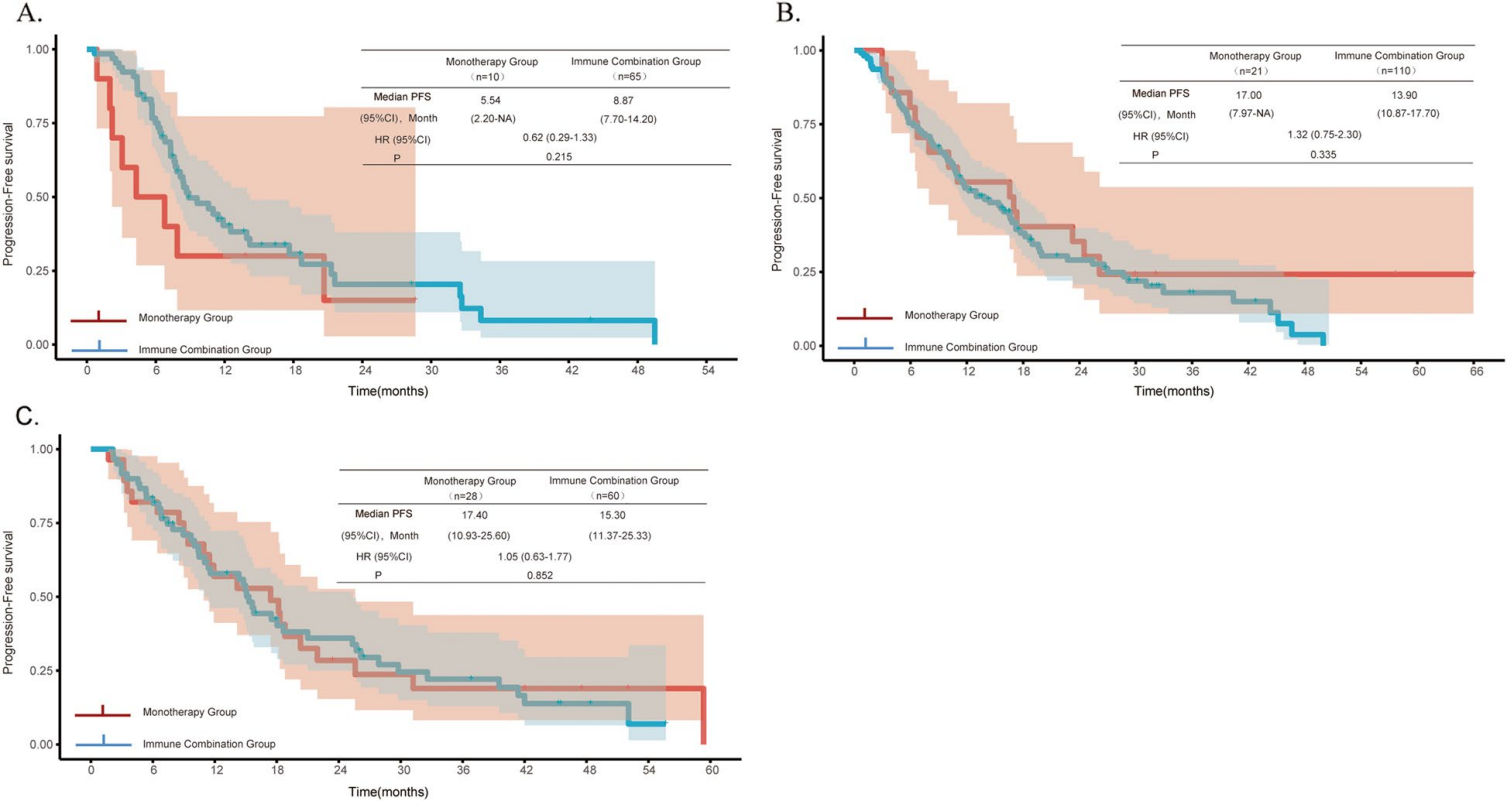
Kaplan–Meier curves showing (**A**) PFS in patients with PD-L1 expression < 1%, (**B**) PFS in patients with PD-L1 expression between 1%−49%, and (**C**) PFS in patients with PD-L1 expression ≥ 50%

In the subgroup analysis of OS, we further analyzed the efficacy of the drugs after administration based on age (< 75 years and ≥ 75 years) and PD-L1 expression levels. The results showed that in patients with negative PD-L1 expression, the median OS in the immune combination group was 31.53 months (95%CI 28.27-NA), which was superior to that in the monotherapy group of 11.42 months (95%CI 6.77-NA, P = 0.043). In patients with PD-L1 expression levels of 1%−49%, the median OS in the immune combination group was 46.03 months (95%CI 26.63-NA), while that in the monotherapy group was 29.27 months (95%CI 17.00-NA), and no significant difference was observed between the two groups (HR = 0.81, 95%CI 0.39–1.67, P = 0.569). Similarly, in patients with PD-L1 expression levels ≥ 50%, the median OS in the immune combination group was 41.83 months (95%CI 27.87-NA), and that in the monotherapy group was 28.40 months (95%CI 14.23-NA), and no significant difference was observed between the two groups either (HR = 0.63, 95%CI 0.33–1.21, P = 0.161) (Figs. 3). As for PFS, we found that regardless of the expression level of PD-L1, there was no significant difference in PFS between the two groups (Fig. 4).

**Fig. 5:**
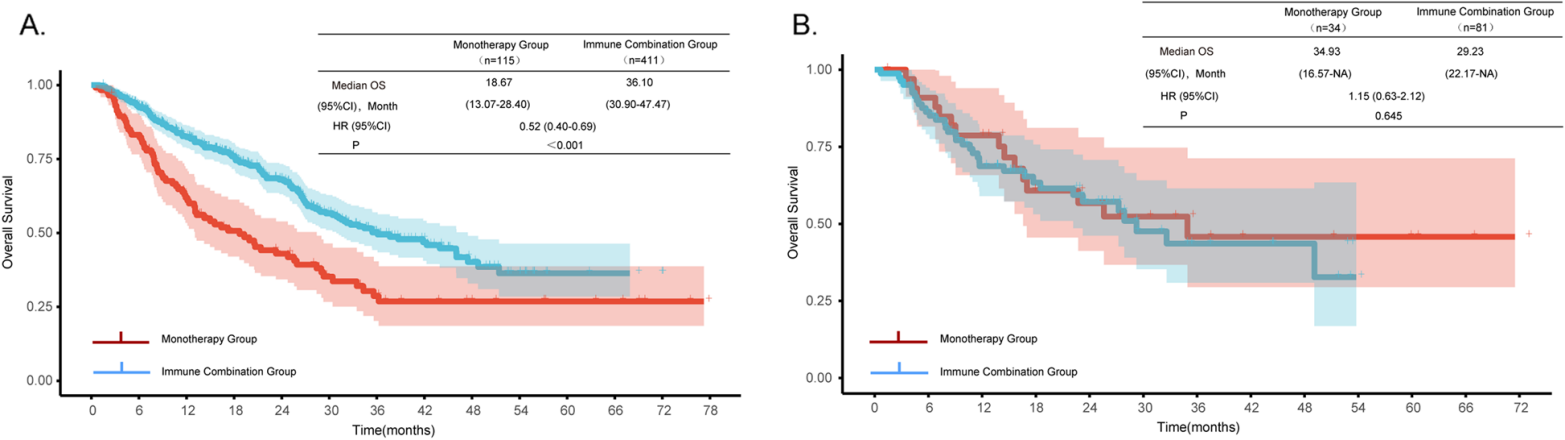
Kaplan–Meier curves showing (**A**) PFS in patients aged < 75, and (**B**) PFS in patients aged ≥ 75

**Fig. 6:**
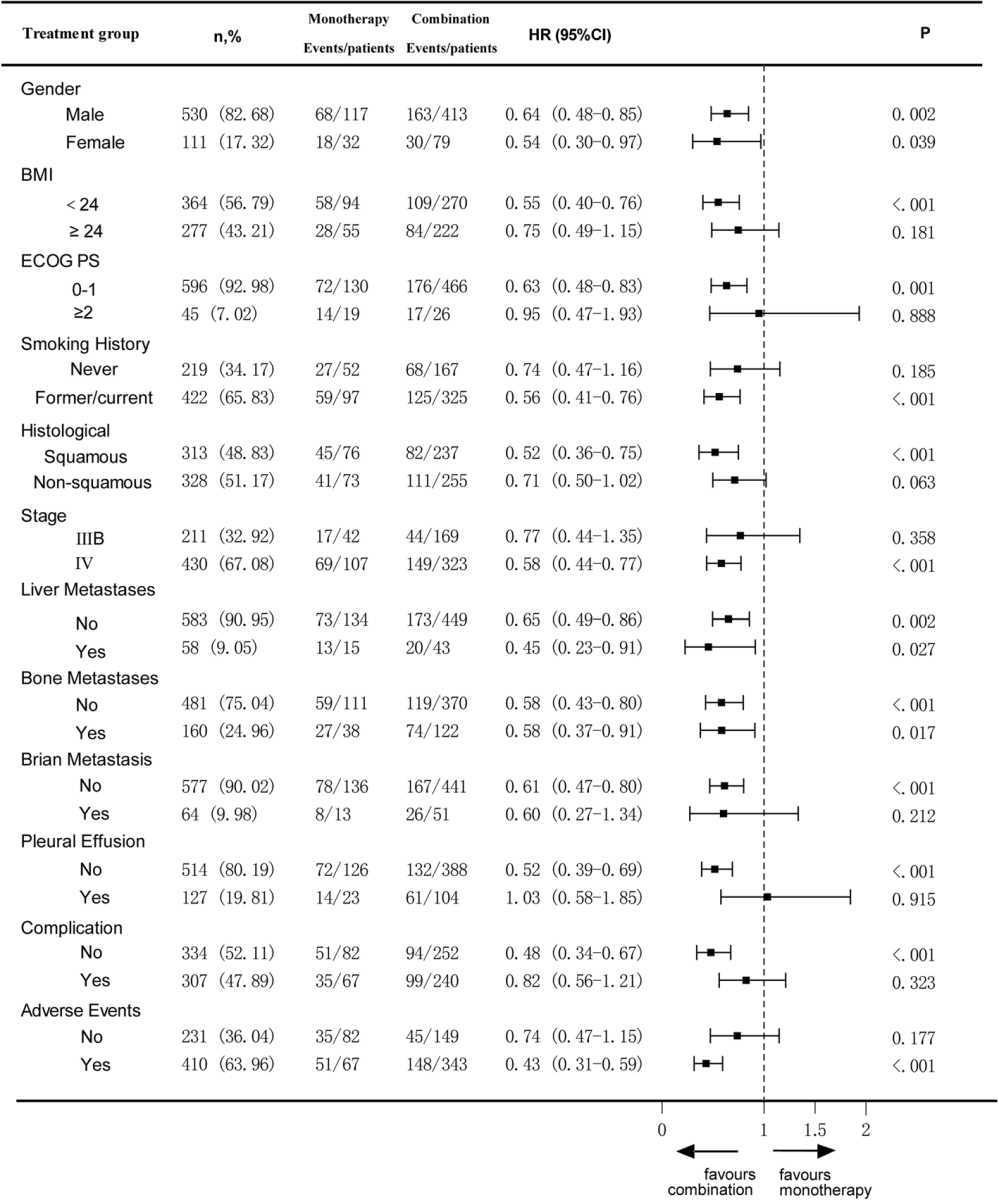
Subgroup analysis of OS based on different characteristics

In the stratified analysis by age, it was found that among patients aged < 75 years (*n* = 526), the OS of the immune combination group was 36.10 months (95%CI 30.90–47.47), which was significantly better than that of the monotherapy group at 18.67 months (95%CI 13.07–28.40, P < 0.001). However, in the ≥ 75-year subgroup (*n* = 115), combination therapy showed no OS advantage (29.23 vs. 34.93 months, *P* = 0.645), though limited sample size precludes definitive conclusions. (Fig. 5). In addition, the median OS was significantly prolonged in patients with BMI < 24 kg/m^2^ (HR = 0.55, 95%CI 0.40–0.76, *P* < 0.001), ECOG PS score between 0 and 1 (HR = 0.63, 95%CI 0.48–0.83, *P* = 0.001), a history of smoking (HR = 0.56, 95%CI 0.41–0.76, *P* < 0.001), squamous cell carcinoma (HR = 0.52, 95%CI 0.36–0.75, *P* < 0.001), stage IV disease (HR = 0.58, 95%CI 0.44–0.77, *P* < 0.001), no brain metastasis (HR = 0.61, 95%CI 0.47–0.80, *P* < 0.001), no malignant pleural effusion (HR = 0.52, 95%CI 0.39–0.69, P < 0.001), and no comorbidities (HR = 0.48, 95%CI 0.34–0.67, *P* < 0.001) (Figs. 6).

### Univariate and multivariate analyses

This study evaluated the clinical factors related to the prognosis of elderly patients with advanced NSCLC through univariate and multivariate Cox proportional hazards regression models. The results of the univariate analysis showed that ECOG PS score ≥ 2 (HR = 2.78, 95%CI 1.91–4.05, *P* < 0.001), stage IV disease (HR = 2.12, 95CI 1.59–2.81, *P* < 0.001), liver metastasis (HR = 1.97, 95%CI 1.37–2.84, *P* < 0.001), bone metastasis (HR = 2.22, 95%CI 1.74–2.83, P < 0.001), malignant pleural effusion (HR = 1.89, 95%CI 1.45–2.46, *P* < 0.001), and combined treatment (HR = 0.62, 95CI 0.48–0.79, *P* < 0.001) were all significantly associated with OS. After adjusting for confounding variables through multivariate analysis, it was found that ECOG PS score ≥ 2 (HR = 1.87, 95%CI 1.26–2.79, P = 0.002), liver metastasis (HR = 1.62, 95%CI 1.11–2.36, *P* = 0.013), bone metastasis (HR = 1.84, 95%CI 1.40–2.42, *P* < 0.001), malignant pleural effusion (HR = 1.64, 95%CI 1.24–2.16, P < 0.001), and combined treatment (HR = 0.54, 95CI 0.41–0.72, *P* < 0.001) remained independent predictors of OS. The results indicated that combined treatment showed a significant protective effect in the multivariate model, with a 46% reduction in the risk of death compared to the single-agent treatment group (Table 3).

**Table 3:** Univariate and multivariate analyses of prognostic factors for NSCLC patients

Variables	Univariate analysis			Multivariate analysis		
	**HR**	**95%CI**	***P* value**	**HR**	**95%CI**	***P* value**
Gender (Female/Male)	1.04	0.76–1.41	0.821			
Age	1.06	0.78–1.45	0.690			
(≥ 75 years/< 75 years)	0.85	0.67–1.08	0.191			
BMI (≥ 24/< 24)	0.85	0.67–1.09	0.208			
Smoking status	1.07	0.85–1.36	0.548			
(Never/Former or Current)	**2.78**	**1.91–4.05**	** < 0.001**	**1.87**	**1.26–2.79**	**0.002**
Stage (IV/IIIB)	**2.12**	**1.59–2.81**	** < 0.001**			
Liver metastases (Yes/No)	**1.97**	**1.37–2.84**	** < 0.001**	**1.62**	**1.11–2.36**	**0.013**
Bone metastases (Yes/No)	**2.22**	**1.74–2.83**	** < 0.001**	**1.84**	**1.40–2.42**	** < 0.001**
Brain metastases (Yes/No)	1.31	0.92–1.88	0.140			
Pleural Efusion (Yes/No)	**1.89**	**1.45–2.46**	** < 0.001**	**1.64**	**1.24–2.16**	** < 0.001**
Complication (Yes/No)	0.98	0.78–1.24	0.880			
Combined (Yes/No)	**0.62**	**0.48–0.79**	** < 0.001**	**0.54**	**0.41–0.72**	** < 0.001**

### Safety

In terms of safety, the immune combination group showed a significantly increased risk of toxicity compared with the monotherapy group. In the overall population, the incidence of any-grade AEs in the immune combination group was 69.72% (343 cases), significantly higher than that in the monotherapy group, which was 44.97% (67 cases) (*P* < 0.001). Specifically, the incidence of grade 1–2 AEs in the immune combination group was higher than that in the monotherapy group (63.21% vs 40.29%, *P* < 0.001), and the incidence of grade 3–4 AEs was significantly different by 2.45 times (16.46% vs 6.71%, *P* = 0.003) (Table 4). Among patients aged ≥ 75 years, 15 cases (44.12%) in the immune combination group and 46 cases (56.79%) in the monotherapy group experienced AEs, among which the incidence of high-grade AEs was higher in the immune combination group (1.94% vs 16.05%, *P* = 0.037) (Table 5). The above results indicate that although combination therapy may improve efficacy, its toxicity risk should be closely monitored.

**Table 4:** Adverse events in the overall population

AEs	Monotherapy Group n = 149	Immune combination Group n = 492	*P* value
Any grade	67 (44.97%)	343 (69.72%)	< 0.001
Grade 1–2	60 (40.29%)	311 (63.21%)	< 0.001
Grade 3–4	10 (6.71%)	81 (16.46%)	0.003

*AEs* adverse events

**Table 5:** Adverse events in people aged ≥ 75 years

AEs	Monotherapy Group n = 34	Immune combination Group n = 81	*P* value
Any grade	15 (44.12%)	46 (56.79%)	0.214
Grade 1–2	15 (44.12%)	40 (49.38%)	0.606
Grade 3–4	1 (1.94%)	13 (16.05%)	0.037

*AEs* adverse events

**Fig. 7:**
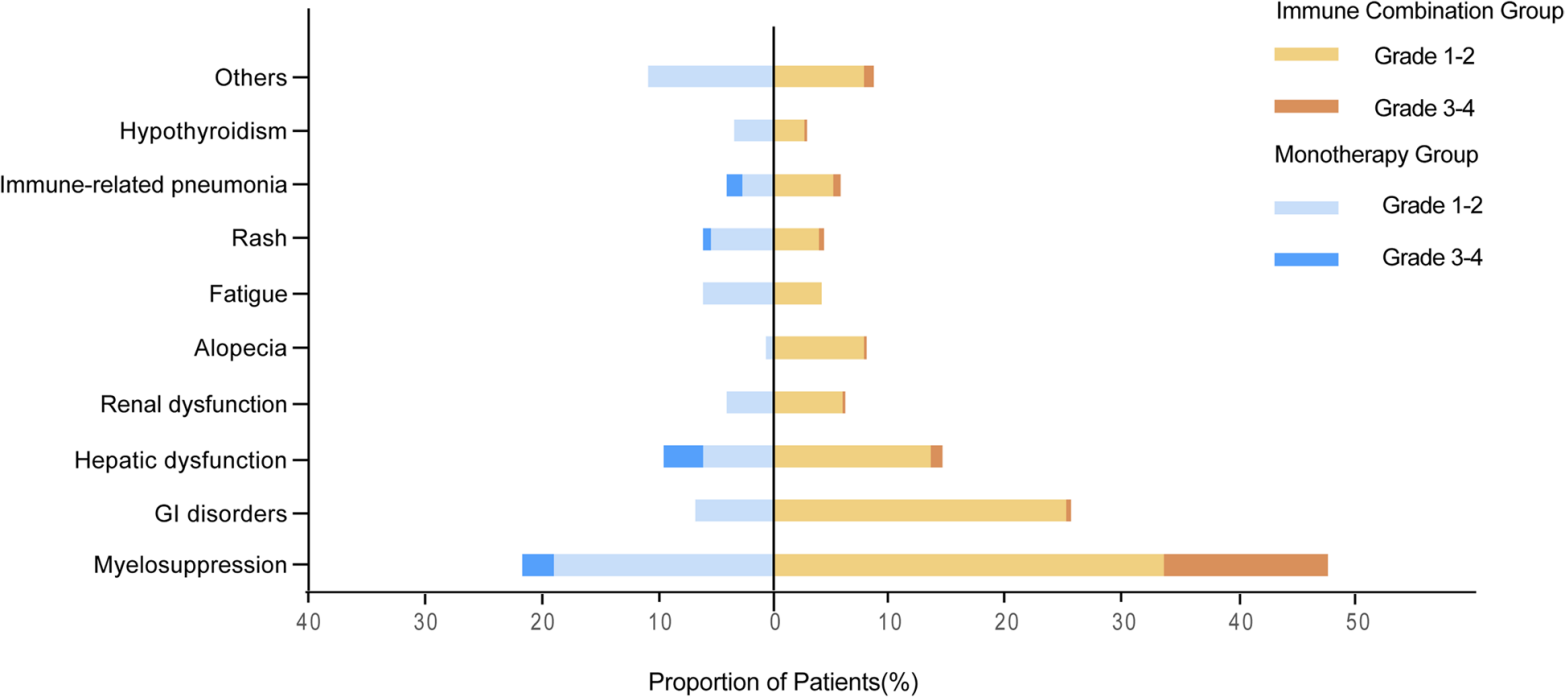
Treatment-related adverse events

Further analysis of specific AEs types (Fig. 7) revealed that the incidence of myelosuppression in the immune combination group was significantly higher than that in the monotherapy group, and most were grade 1–2. Meanwhile, the incidence of gastrointestinal adverse events, liver function abnormalities, and alopecia in the immune combination group was also higher than that in the monotherapy group. Regarding grade 3–4 AEs, the monotherapy group mainly included myelosuppression, liver function abnormalities, immune-related pneumonia, and rash; in addition to these, the immune combination group also presented with high-grade AEs such as digestive tract reactions, renal function abnormalities, alopecia, and hypothyroidism.

### Prognosis nomogram for NSCLC patients

**Fig. 8:**
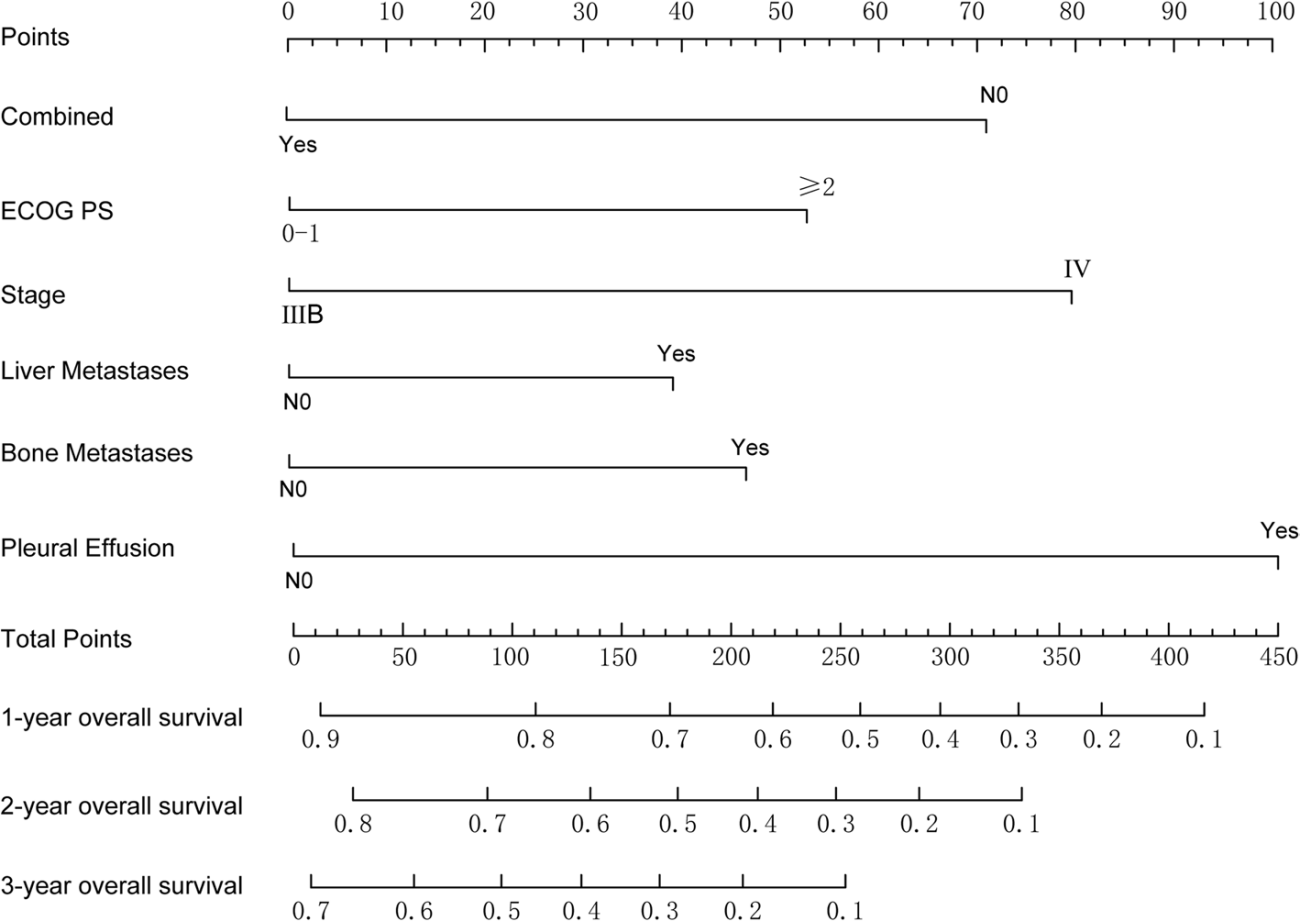
The construction of the nomogram for predicting the 1-year, 2-year, and 3-year OS

To accurately predict the OS of elderly patients with advanced NSCLC after immunotherapy, this study utilized LASSO regression analysis to select key variables and constructed a nomogram risk prediction model. This model integrates six key prognostic factors, including whether combined chemotherapy was received, ECOG PS score, the presence of distant metastasis, the presence of liver metastasis, the presence of bone metastasis, and the presence of pleural effusion. Based on these factors, we developed a prognostic nomogram model for OS in elderly patients with advanced NSCLC after immunotherapy (Fig. 8). This model aims to provide clinicians with an intuitive and quantitative tool to facilitate the assessment of patients'prognostic risks.

**Fig. 9:**
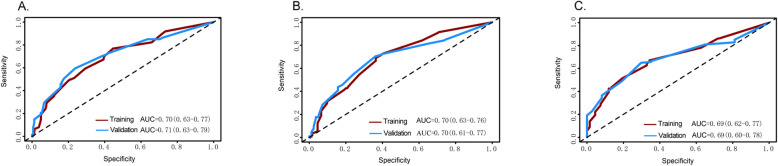
The ROC curves of the training set and validation set for the prediction models of (**A**) 1-year survival rate; (**B**) 2-year survival rate; (**C**) 3-year survival rate

The ROC curve is a key indicator for evaluating the discrimination ability of predictive models. The closer the AUC value is to 1, the stronger the discrimination ability of the model. In the training cohort, the AUC values for predicting 1-year, 2-year, and 3-year overall survival were 0.70, 0.70, and 0.69, respectively. In the internal validation cohort, the corresponding AUC values were 0.70, 0.71, and 0.69 (Fig. 9). These results indicate that the model demonstrates good discrimination ability for 1-year and 2-year predictions, but its predictive performance for 3-year survival is somewhat insufficient.

**Fig. 10:**
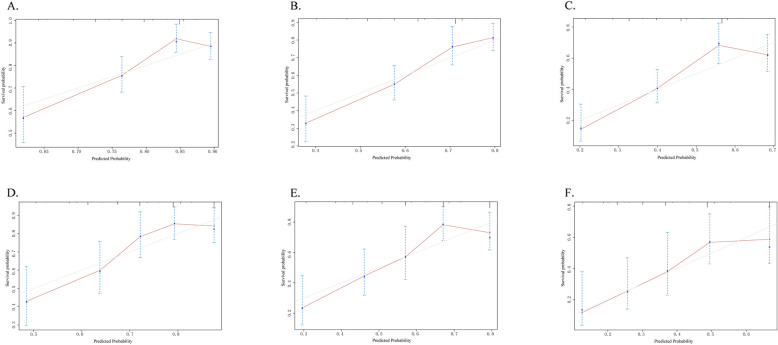
Calibration curves for predicting the 1-year, 2-year, and 3-year OS in the training cohort (**A**, **B** and **C**). Calibration curves for predicting the 1-year, 2-year, and 3-year OS in the internal validation cohort (**D**, **E**, and **F**)

Further calibration curve analysis revealed that the nomogram prediction model for elderly patients with advanced lung cancer had a relatively high prediction accuracy for 1-year survival rate, with the predicted probability being consistent with the actual survival rate. However, as the prediction time was extended (2-year and 3-year survival rates), the prediction accuracy of the model decreased (Fig. 10).

**Fig. 11:**
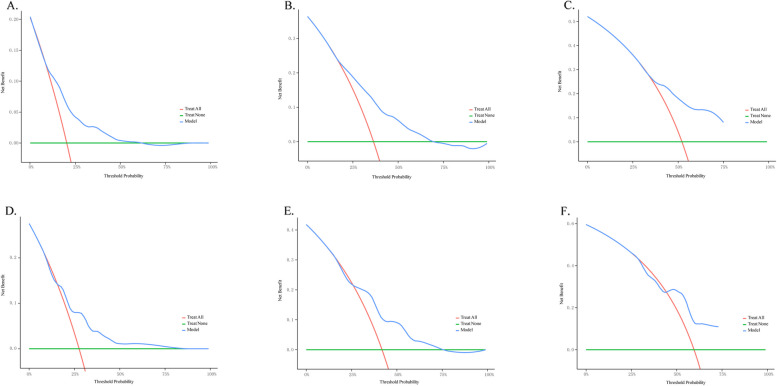
Decision curves for predicting the 1-year, 2-year, and 3-year OS in the training cohort (**A**, **B** and **C**). Calibration curves for predicting the 1-year, 2-year, and 3-year OS in the internal validation cohort (**D**, **E**, and **F**)

In addition, this study also plotted the DCA curve and found that the model demonstrated a relatively good net benefit in the prediction of a 1-year survival rate, especially within the lower risk threshold range. In the prediction of 2-year and 3-year survival rates, the model still showed a certain net benefit advantage within the medium risk threshold range, but the net benefit decreased at higher risk thresholds (Fig. 11). This indicates that the model's predictive performance is relatively reliable in the short term, but it may need further optimization for long-term survival prediction.

## Discussion

In recent years, with the acceleration of global population aging, the incidence of lung cancer among the elderly has continued to rise. As this group ages, their physiological functions gradually decline and multiple organ functions decrease, leading to reduced drug tolerance and varying degrees of impact on the efficacy of immunotherapy. Currently, research on lung cancer in elderly patients is relatively scarce, resulting in insufficient representation of this group in clinical trials and presenting numerous challenges for clinical treatment.

### Efficacy of combination therapy vs. monotherapy in elderly patients

This study systematically evaluated the efficacy and safety of immune monotherapy versus immune combination chemotherapy in elderly patients with advanced NSCLC. The results showed that the median OS in the immune combination group was significantly longer than that in the monotherapy group by 14.84 months (35.37 vs. 20.53 months, HR = 0.62, *P* < 0.001). This finding is consistent with the TORG1630 study [18] , which demonstrated that the OS of nivolumab combined with chemotherapy was significantly better than that of nivolumab monotherapy, especially for patients over 65 years old (HR = 0.41, 95%CI 0.23–0.71). Another meta-analysis [19] included 11 articles comparing immune combination chemotherapy with immune monotherapy and found that the efficacy of immune combination chemotherapy was significantly better than that of immune monotherapy regardless of the expression level of PD-L1. These studies suggest that the survival advantage of combination therapy may be attributed to the synergistic mechanism of chemotherapy and immunotherapy. Chemotherapy drugs can induce immunogenic cell death in tumor cells, release a large amount of tumor-associated antigens, and promote the maturation and antigen presentation of dendritic cells (DC). At the same time, chemotherapy can also reshape the immune microenvironment by depleting immunosuppressive cells, and enhancing T cell infiltration and function, and this"immune priming"effect is particularly significant in PD-L1 negative patients [20] . However, in this study, no significant difference was observed in PFS between the combination immunotherapy group and the single-agent group (11.87 months vs 10.67 months, *P* = 0.535). The research by Satoshi Endo's team [21] also found no significant difference in median PFS between the combination therapy group and the immunotherapy monotherapy group, and the same conclusion was drawn by Lingling Li's [22] meta-analysis, which is consistent with this study. However, in the study by Chih-Hsi Scott Kuo's team [23] , it was found that the median PFS of the combination of immunotherapy and chemotherapy was superior to that of immunotherapy monotherapy. The possible reason for this might be that all the patients included in this study were elderly, and thus, considering their physical condition, comorbidities, and tolerance to chemotherapy, they might have received low-dose chemotherapy regimens or had their doses reduced or treatment interrupted due to toxicity during the treatment process, resulting in limited synergy with immunotherapy.

### PD-L1 expression and age-stratified outcomes

PD-L1 expression is one of the important biomarkers for immunotherapy in advanced non-small cell lung cancer, and its expression level can affect treatment selection and clinical outcomes [24] . Patients with high PD-L1 expression are generally considered to have stronger immunogenicity. Immune combination therapy can enhance the efficacy of immunotherapy by releasing tumor immunogenicity, regulating the tumor microenvironment, and accelerating drug delivery [25]. Among them, the most commonly used mode is the combination of immunotherapy and chemotherapy, and the application of this mode is not limited by the expression level of PD-L1 [26] .

The stratified analysis based on the expression level of PD-L1 in this study showed that in patients with negative PD-L1 expression, the combination of immunotherapy and chemotherapy had a significant survival benefit compared with immunotherapy alone (31.53 months vs. 11.42 months, *P* = 0.043), while in patients with PD-L1 expression levels between 1 and 49% (46.03 months vs. 29.27 months, *P* = 0.569) or ≥ 50% (41.83 months vs. 28.40 months, P = 0.161), there was no significant difference in efficacy between the combination of immunotherapy and chemotherapy and immunotherapy alone. However, Hong's team [27] found that for patients with PD-L1 expression levels over 50%, the combination of pembrolizumab and chemotherapy achieved better OS compared with immunotherapy alone. Unfortunately, this study did not conduct stratified analysis by age. Additionally, several studies [8, 9, 28] have indicated that for patients with negative PD-L1 expression, expression levels between 1 and 49%, or over 50%, the combination of immunotherapy and chemotherapy is associated with better survival outcomes. In addition, there was no significant difference in PFS between the two groups regardless of PD-L expression. The team led by Yasuyuki Ikezawa [29] found that the combination of immunotherapy and chemotherapy could significantly prolong the median PFS of patients with high PD-L1 expression, which was superior to immunotherapy alone. Kenji Morimoto et al. [30] also pointed out that combined treatment had a significant advantage over immunotherapy alone in terms of PFS in advanced NSCLC patients with high PD-L1 expression and poor ECOG PS. All these indicate that the specific characteristics of elderly patients may weaken the survival advantage of combined treatment. However, a meta-analysis[31] showed that in patients with PD-L1 ≥ 50%, combined treatment significantly improved PFS and ORR; in patients with PD-L1 between 1 and 49%, combined treatment had a greater advantage in OS. Due to the small sample size and the inclusion of elderly patients in this study, the analysis results may be biased. Further exploration is needed in the future.

Age-stratified analysis revealed that patients under 75 years old in the immune combination group had a more significant benefit compared to the immune monotherapy group (36.10 months vs. 18.67 months, *P* < 0.001), while in patients aged 75 and above, there was no significant difference in OS between the two groups (34.93 months vs. 28.23 months, *P* = 0.645). Yoko Tsukita’s team [32] found that in patients aged 75 and above, the combination of ICIs and chemotherapy did not improve survival compared to ICIs monotherapy. Yasuyuki Ikezawa’s team [33] also found that in this age group, there was no difference in OS between ICIs monotherapy and ICIs combined with chemotherapy, which is consistent with the results of this study. In terms of mechanism, T-cell function and antigen-presenting capacity decline in elderly patients aged 75 and above, leading to a weakened synergistic effect of the immune system with chemotherapy [34] . Immunosenescence may weaken the chemotherapy-induced immunogenic cell death (ICD) effect, making it difficult for combination therapy to effectively activate anti-tumor immune responses. Additionally, the exhaustion of CD8 + T cells and an increased proportion of regulatory T cells (Tregs) may further limit the potential of combination therapy to enhance efficacy [35] . The tumor microenvironment (TME) in elderly patients may be more immunosuppressive [36]. For example, PD-L1 expression may dynamically change with age, with a lower proportion of patients having high expression, making it difficult for combination therapy to enhance immune responses through chemotherapy [37] . Elderly patients often have comorbidities (such as heart and lung dysfunction, and reduced kidney function), and their ability to metabolize and clear chemotherapy drugs is decreased. Side effects of platinum-based chemotherapy, such as bone marrow suppression and nephrotoxicity, may exacerbate organ damage and offset the survival benefits of immunotherapy [38] . Therefore, for elderly patients with advanced NSCLC aged 75 and above, it is necessary to strictly evaluate the patient's PD-L1 expression level, physical condition (such as PS score), organ function, and comorbidities, and closely monitor adverse reactions to select the appropriate treatment plan.

Additionally, this study found that although there was no significant difference in PFS between the two treatment regimens in stage IIIB and IV patients, the OS of stage IV patients treated with immune combination chemotherapy was significantly better than that of the immune monotherapy group. In the IMpower150 trial [39] , atezolizumab combined with chemotherapy and bevacizumab showed a significant survival advantage in stage IV patients, which is consistent with the results of this study. Stage IV patients usually have a higher burden of metastatic tumors and more extensive heterogeneity. Chemotherapy may enhance the long-term effect of immunotherapy by releasing a large amount of tumor antigens to increase immunogenicity and regulate the immunosuppressive microenvironment [40] .

### Safety and tolerability in elderly patients

In terms of safety, the incidence of any-grade AEs in the immune combination group was significantly higher than that in the monotherapy group in the overall population (69.72% vs. 44.97%, *P* < 0.001), and the incidence of grade 3–4 AEs was also higher in the immune combination group (16.46% vs. 6.71%, *P* = 0.003). Subgroup analysis of patients aged ≥ 75 years further revealed the risk of combination therapy, with a higher incidence of grade 3–4 AEs (16.05% vs. 1.94%, *P* = 0.037). This suggests that while combination immunotherapy enhances anti-tumor efficacy, it may also lead to toxicity accumulation due to the synergistic effects of multiple mechanisms, and the risks and benefits need to be weighed in clinical decision-making. Combination immunotherapy enhances anti-tumor effects by synergistically activating the immune system, but it may also trigger more extensive immune activation, leading to damage to normal tissues. In multiple clinical studies [41, 42], the incidence of AEs in the combination of ICIs and chemotherapy was higher than that in ICIs monotherapy. Additionally, a Meta-analysis [43] showed that the relative risk of grade 3–4 AEs in the combination therapy group was significantly higher than that in the monotherapy group (RR = 1.32), and the incidence of fatal AEs (such as myocarditis and colitis) was also higher. Moreover, combination therapy may exacerbate toxicity through mechanisms such as cytokine storm or excessive T-cell infiltration. However, there is relatively limited data on the incidence of AEs in the elderly population. The study by Yoko Tsukita's team [32] found that for patients aged ≥ 75 years, the incidence of high-grade AEs in combination therapy was higher than that in single immunotherapy, which is consistent with the conclusion of this study. A retrospective study by Hayashi's team [44] showed that in elderly patients with advanced lung cancer aged 70 years and above, the frequency of other AEs such as pneumonia, fatigue, rash, diarrhea, and vomiting was higher, and most were mild to moderate grade 1–2 AEs, which is similar to the results of this study. Although the toxicity in the combination therapy group in this study was slightly higher, most were low-grade AEs and were generally acceptable. In addition, some studies [45, 46] have shown that the incidence of hyperprogression after immune monotherapy is higher than that after combination immunotherapy, especially in patients aged ≥ 65 years, with gene mutations, and local recurrence. Therefore, ICIs combined with chemotherapy can be a treatment option for patients aged 65–74 years, but caution should be exercised in patients aged ≥ 75 years.

### Limitations and future directions

However, this study also has some limitations. Firstly, in this study, the sample size of patients aged ≥ 75 years was relatively small, which might lead to a reduction in statistical power and make it difficult to identify real differences in efficacy or safety. And the distribution of stages was not clear, PD-L1 data was missing, and the details of treatment regimens were insufficient, which may mask the impact of treatment heterogeneity on the toxicity and efficacy in elderly patients. Secondly, PD-L1, as a key predictive biomarker for immunotherapy, is closely related to the efficacy of ICIs. In this study, PD-L1 expression was not detected in some patients, which may lead to the results not reflecting the true situation of the overall population. It is hoped that future prospective, multicenter, large-sample studies will further verify this. Thirdly, this study did not assess mutations in immunotherapy-related genes due to the retrospective nature of the analysis and the lack of standardized testing protocols for these biomarkers during the study period. The absence of data on mutations such as TP53 or KRAS limits our ability to explore their potential interactions with immunotherapy efficacy. Emerging evidence suggests that KRAS mutations may modulate PD-L1 expression and influence immunotherapy responses, underscoring the need for prospective studies integrating comprehensive genomic profiling in elderly NSCLC cohorts[47]. Finally, the prognostic model has only undergone internal validation and lacks validation from an external independent cohort, which limits the clinical promotion value of the model.

In the future, prospective studies need to be conducted, integrating multiple omics biomarkers, such as TMB, dynamic changes in peripheral blood immune cells, etc., and geriatric functional assessment tools, to establish individualized treatment decision-making models and provide a more solid theoretical basis and practical guidance for the clinical treatment of elderly lung cancer patients. And future research should expand the scale of prospective studies, further subdivide age subgroups, and intensify the exploration of efficacy, safety, and biomarkers specific to the elderly population to achieve the goal of precision medicine.

## Conclusion

Real-world data-based studies have shown that the median OS in the immune combination group is significantly better than that in the monotherapy group, and the survival benefit is mainly concentrated in patients under 75 years old. However, the incidence of grade 3–4 AEs in the immune combination group is significantly higher than that in the monotherapy group, especially in patients aged 75 years or older. This suggests that for elderly patients aged 75 years or older, the choice of combination regimens should be made with caution, weighing the efficacy against the risk of toxicity. Prognostic analysis further revealed that ECOG PS ≥ 2, liver metastasis, bone metastasis, and malignant pleural effusion are risk factors for prognosis, indicating that for elderly patients, the baseline status and metastatic burden should be prioritized for assessment. In summary, this study verified the clinical value of immunotherapy plus chemotherapy in some elderly patients, while also revealing the limited tolerance of the elderly population. While combination therapy demonstrates significant survival benefits for patients < 75 years with ECOG PS 0–1 and low metastatic burden, for those ≥ 75 years, treatment decisions should prioritize individualized evaluations of fitness (e.g., ECOG PS, comorbidity burden) rather than universally excluding combination therapy.

## Data Availability

The patient datasets used and/or analyzed during the current study are available from the corresponding author upon reasonable request.
